# P-1938. Impact of Race and Ethnicity on Patient-reported Health and Symptoms Following COVID-19 or Long COVID Diagnosis

**DOI:** 10.1093/ofid/ofae631.2097

**Published:** 2025-01-29

**Authors:** Thomas F Oppelt, Michael Caron, Jamie Zagorski, Rodney H Taylor, Chinonso Akano, Lisa McCorkell, LaKeisha Williams, Gary A Puckrein

**Affiliations:** Gilead Sciences, Inc, Foster City, California; Gilead Sciences, Inc., Foster City, California; Gilead Sciences, Inc., Foster City, California; Gilead Sciences, Inc., Foster City, California; Gilead Sciences, Inc., Foster City, California; Patient-Led Research Collaborative, Oakland, California; Center for Minority Health and Health Disparities Research and Education, Xavier University of Louisiana, New Orleans, Louisiana; National Minority Quality Forum, Washington, District of Columbia

## Abstract

**Background:**

SARS-CoV-2 infection and COVID-19 hospitalization and mortality rates are higher in Black and Hispanic patients compared to White patients. Patient perspectives give key insights into healthcare inequities and treatment disparities. This study aimed to describe differences in the perceptions of health status and COVID-19 symptoms by race and ethnicity.

**Figure:**
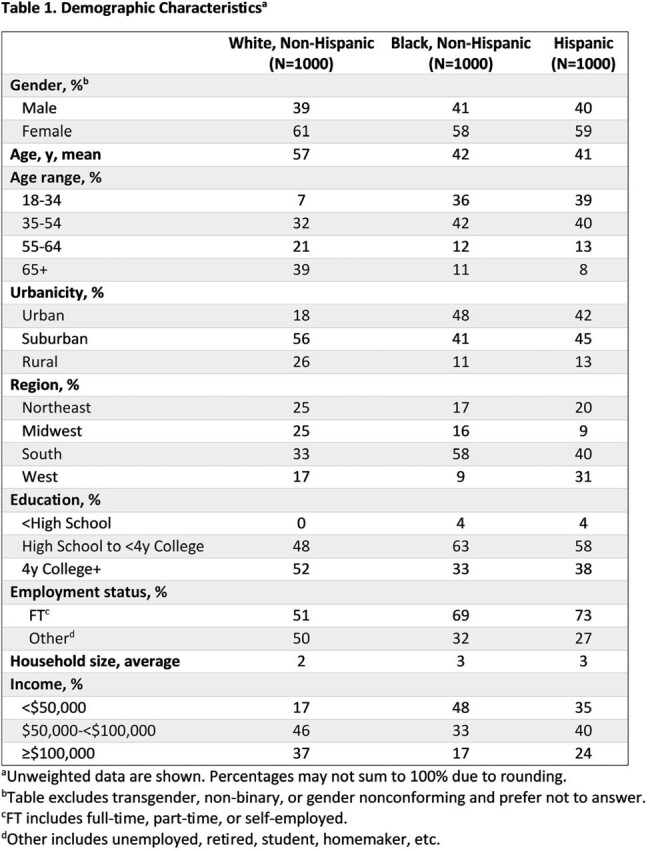

**Methods:**

An online survey was conducted by the Harris Poll in the US (March 12-April 1, 2024) among adults aged ≥18 y diagnosed with, but not hospitalized for, COVID-19 or Long COVID (LC) in the past 12 months. Non-demographic data were weighted by age, gender, race/ethnicity, region, education, marital status, household size, household income, and propensity to be online to align them with their real-world proportions. Outcomes were compared among non-Hispanic White (White), non-Hispanic Black (Black), and Hispanic participants. Statistical significance was determined by two-tailed t-test (95% CI).

**Figure:**
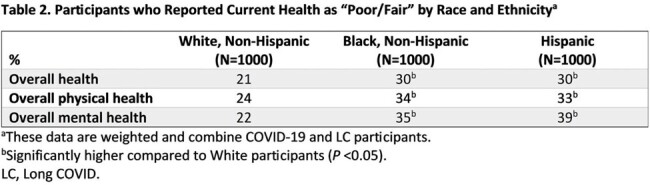

**Results:**

3090 participants diagnosed with COVID-19 or LC in the past 12 months completed the survey, with equal representation of White, Black, and Hispanic participants. White participants were more likely than Black and Hispanic participants to be older, live in suburban areas, have a 4-y college education, and earn >$100,000 (Table 1). Black and Hispanic participants were more likely to live in the South and be employed full-time. More Black and Hispanic participants reported their current health as “Poor/Fair” than White participants (*P* < 0.05; Table 2).

Compared to White participants, greater proportions of Black and Hispanic participants described their initial COVID-19 or LC symptoms as “Very/Somewhat Severe” (White: 40%; Black: 55%; Hispanic: 59%; *P* < 0.05) and reported difficulty breathing, nausea/vomiting, diarrhea, and persistent pain/pressure in the chest (*P* < 0.05; Table 3). More Black (19%) and Hispanic (23%) participants reported that gaining access to healthcare when needed was “Very/Somewhat Difficult” versus White participants (8%; *P* < 0.05).

**Figure:**
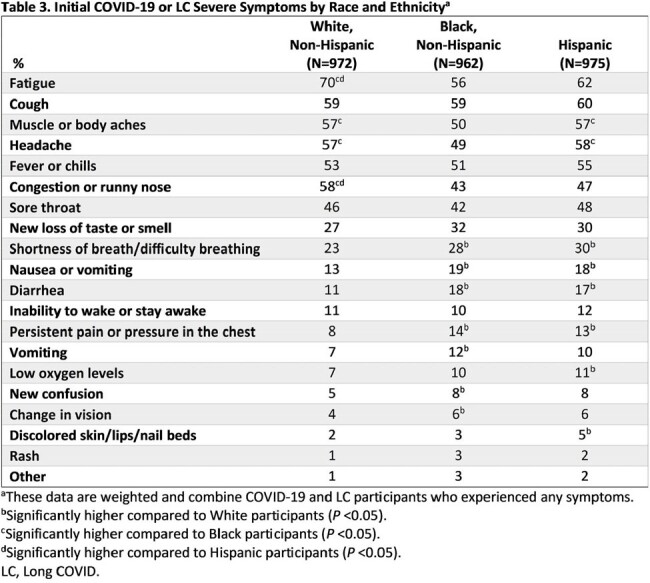

**Conclusion:**

Black and Hispanic participants recently diagnosed with COVID-19 or LC reported poorer health and more severe initial symptoms compared to White participants, which aligns with other studies highlighting healthcare disparities and treatment inequities.

**Disclosures:**

Thomas F. Oppelt, PharmD, BCPS, Gilead Sciences, Inc: I am an employee of Gilead Sciences, Inc|Gilead Sciences, Inc: Stocks/Bonds (Public Company) Michael Caron, PharmD, Gilead Sciences, Inc.: Employee|Gilead Sciences, Inc.: Stocks/Bonds (Public Company) Jamie Zagorski, MSN, FNP, Gilead Sciences, Inc.: Employee|Gilead Sciences, Inc.: Stocks/Bonds (Public Company) Rodney H. Taylor, PharmD, Gilead Sciences, Inc.: Employee|Gilead Sciences, Inc.: Stocks/Bonds (Public Company) Chinonso Akano, PharmD, Gilead Sciences, Inc.: Employee|Gilead Sciences, Inc.: Stocks/Bonds (Public Company) LaKeisha Williams, PharmD, MSPH, Gilead Sciences, Inc.: Grant/Research Support Gary A. Puckrein, PhD, Dexcom: Grant/Research Support|Gilead Sciences, Inc.: Grant support for National Minority Quality Forum|National Minority Quality Forum: Employee

